# Proceedings of the American Dental Association for 1883

**Published:** 1883-10

**Authors:** 


ARTICLE II.

### A SYNOPSIS OF THE PROCEEDINGS OF THE AMERICAN DENTAL ASSOCIATION.

(Held at Niagara Falls, August 7-10 inclusive, 1883.)

The twenty-third annual meeting of the American Dental Association was opened on Tuesday morning, Aug. 7, at 11:00 o'clock A. M., Vice-President, Dr. G. J. Fredericks, of New Orleans, in the chair.

The sessions were held in the parlors of the International Hotel. The chair of the late President, Dr. W. H. Goddard, was vacant and draped in mourning, and a picture of him hung above it.

The roll of qualified members was then called, and about the usual number, at this stage of the proceedings, answered to their names. Three of those whose faces have usually been seen promptly at the beginning were absent because of an imperative call to another and higher sphere of action, viz., Dr. W. H. Goddard, of Louisville, Ky., Dr. Marshall H. Webb, of Lancaster, Pa., and Dr. William H. Allen, of New York. For each of these a committee was appointed to prepare expressions and tribute of appreciation and regard.

The acting President now delivered the annual address.

Amendments to the constitution, which were proposed at the last annual meeting, were now taken up, and the following were adopted, viz: SECTION VII., to read as follows:

"That all resolutions appropriating moneys, except for the legitimate expenses of the Association, shall require a two-thirds vote of all the members present.

A motion to amend ART. V., SEC. IV., was indefinitely postponed.

The Executive Committee recommend that the afternoon and evening be devoted to the work of Sections, and that the regular sessions of this body should be from 9 A. M., to 1:30 P. M., and from 9 P. M., to adjournment.

The recommendation of the Executive Committee was accepted and adopted.

The committee on Credentials reported that sixteen new members were received.

The Publication Committee reported that arrangements had been made by which the printing of the transactions had been done without expense to the Association by the S. S. White Dental Manufacturing Company.

A cordial vote of thanks was given to the Manufacturing Company for the prompt and thorough manner in which they had performed the work of publication of the proceedings.

According to arrangement, the afternoon was to be devoted to Section work, and the presiding officers were requested to call together their respective Sections for the accomplishment of such work as might come before them.

The Association now adjourned till 9 o'clock to-morrow morning.

SECOND DAY—MORNING SESSION.

The meeting was called to order by the acting President, Dr. J. G. Fredericks, at 9:30 A. M. The minutes of the session of the first day were read and approved.

The committee appointed to report upon the death of Dr. Webb, presented the following, which was read by Prof. Peirce, of Philadelphia.

This Association desires permanent record made of the loss incurred by it and by the dental profession, in the death of our late member, Dr. Marshall H. Webb, whose decease occurred since our last meeting.

Dr. Webb was possessed of such manipulative skill as has been attained by very few dental practitioners. His prominence in this respect which was almost universally conceded, was not so much the result of superior natural gifts as of careful concentration and persistence in efforts to excel. He was not content unless in every operation he realized his ideal; not only earnest and conscientious, but ambitious. He was not satisfied to work as well as the best, not content to outdo all others, but strove always to improve on his own performance, constantly advancing his standard of requirement. There was however no element of selfishness in his professional labors; his was not an ignoble strife for leadership. He was ready and willing to tell to any student or practitioner the methods by which he reached his results; not only ready and willing, but eager to show all the steps of an operation, and never so happy as when he succeeded in awakening or stimulating an ambition in others to acquire a skill equal to his own. In this manner he did much to improve the quality of service in very many dental offices throughout the country. He has left many followers whose first ideas of thoroughness in dental operations date from the time of their witnessing a clinic by Dr. Webb; these have in their time become centres from which like influences are emanating, and thus, though resting from his labors, " his works do follow him."

*Resolved*, That a copy of the foregoing expressions of our loss and of our appreciation of Dr. Webb's worth be inscribed upon a memorial page in the transactions, and that a copy also be sent to the family of our departed friend.

C. N. PEIRCE,
E. T. DARBY,
S. J. PERRY,
Committee.

After the reading of the report, Dr. Taft said he could not let the occasion pass without adding a word to the very excellent report just read. He referred to the remarkable ability of Dr. Webb as an operator, one who had made attainments not excelled if equalled by any of his age. His special natural endowments coupled, with unwonted dovotion, industry, and perseverance, enabled him to attain his high point of excellence. In respect to these attainable characteristics he is a shining example for the young men of the profession. He labored not so much for his own interest as for that of his patients and the profession. To the latter he was always willing, and even desirous to communicate, whatever of principles and methods he possessed, that might aid him in their work. In this respect he was an example to us all.

His effort was an unselfish one to build up the profession. Indeed, he thought too little of his own interests both physical and financial. He has left a written record of what he accomplished, and the methods he employed, which have been published, will be valuable to any student and practitioner as well. It should be in the hands of every dentist in the country.

Section 6 was now called, when Dr. Odell, of New York, chairman of the section, said that there were two papers to be read from this section, whereupon Dr. A. G. Frederichs, of New Orleans, read a paper on " Syphilitic Teeth."

The importance and difficulty of making a correct diagnosis of syphilitic cases were made a prominent point. The paper put a large discount upon the claims made so frequently for the definite markings upon the teeth by syphilis.

The subject elicited considerable discussion in which Drs. Atkinson, Peirce, Darby, Kingsley, Abbott, and Park took part.

Dr. Harlan then read a paper on "Pyorrhœa Alveolaris," which elicited much interest, and was discussed by Drs. Bodecker, Darby, Abbott, Wilson, Atkinson, and Taft.

Section 6 was then passed.

Section 5 was then called; when Dr. Bodecker read a paper on "The action of Arsenious Acid upon Dentine and Pulp Tissue."

This was an exceedingly interesting paper, which presented some facts, hitherto not recognized, that should receive the earnest attention of those who extensively use arsenious acid in the treatment of sensitive dentine and exposed pulps.

Dr. Bodecker made a clear illustration of the facts given in his paper with the microscope and black-board. The subject was discussed by Drs. Abbott, Taft, Buckingham, Brown, Park, and Richardson, of London. After which the section was passed.

Section 7 being called, Dr. Barrett made an interesting report on "Physiology and Etiology," which quite well illustrated the fact that rapid progress is being made on these subjects in the dental profession.

Dr. Peirce, chairman of the Committee on Prizes, stated that the committee had in its possession a paper which was intended for a prize. It was a very good paper; would require about an hour for its reading.

It was moved to refer the paper to Section 7, where the mover supposed it belonged.

The motion was quite freely discussed by several members, after which Dr. Peirce said that it was the committee's privilege to withhold the paper until next year, which it had decided to do.

Dr. Buckingham read his report, as chairman of Section 1, in which he stated that his section had no papers to present, but he would now present some thoughts on chemistry.

The subject of "Prosthetic Dentistry" was now taken up and discussed (there being no paper on it) by Drs. W. H. Truman, Stockton, Bodecker, Priest, and Mattison. The section was passed.

Dr. Edwin Richardson, of London, England, being present, was introduced and tendered the privilege of the floor.

He expressed thanks for the honor thus conferred, and said he came rather to learn than to speak.

Miscellaneous business was new taken up. Dr. Odell offered the following:

*Resolved,* That hereafter the calling of the sections shall be in regular progressive numerical order, and that in case any section is not ready to report when called, such section shall be passed without recall until it again comes up in its regular order.

Adopted.

The following request was made by the Association, viz: That the exhibitors of dental instruments and supplies close their doors and keep them closed during the sessions of this body. This was cordially complied with.

Adjourned till 8 o'clock P. M.

SECOND DAY—EVENING SESSION.

At 8:30 the Association was called to order, President Frederichs in the chair. The minutes of the morning session were read and approved.

Dr. Peirce reported for the Committee on Prize Essay and after some discussion the report was adopted.

A motion was then made that the election of officers and the selection of the place for holding the next meeting take place to-morrow evening, beginning at 8 o'clock. Approved.

Dr. N. W. Kingsly was called upon for remarks upon a subject upon which he has bestowed much thought and study, and upon which he has had a large range of experience.

By the use of illustrations upon the black-board, and by very clear descriptions, he made the subject of "Articulate Speech" very interesting. He presented some ideas and thoughts new to most of those present.

Adjourned till 9 o'clock A. M. to-morrow.

THIRD DAY—MORNING SESSION.

The Association was called to order at 9 o'clock, President Fredericks in the chair.

The minutes of yesterday's session were read and approved.

The Committee on Prize Essay presented the following report:

"Your committee to whom was assigned the duty of deciding upon the merits of essays upon "The Etiology of Dental Caries," offered for a prize $200, which was last year appropriated by this Association for the purpose, would respectfully report that but one essay has been received, and that from the hands of Dr. W. D. Miller, of Berlin, Germany. The committee have carefully read this, and while the views contained therein are not original, many of his experiments, which are in detail and made for the purpose of confirming his theory, have not been previously published. Your committee would, therefore, in view of the original work which the author has prosecuted the past two years, the results of which are given in the paper, award to the essayist the $200 appropriated for the purpose."

The regular order was now resumed. and Section 1 was called, viz.: "Prosthetic Dentistry, Chemistry, and Metallurgy.

There was no report, but the subject was discussed by Drs. Watkins, Stockton, Harron, and Morrison. Dr. E. P. Brown illustrated a method of making and inserting artificial teeth, which he had devised and used with great satisfaction. Drs. Buckingham and How, of Philadelphia, discussed the subject of artificial crowns, after which the subject was passed.

Section 2 was called.

Dr. Shepard, of Boston, was absent and Dr. Peirce read the report of the section, stating that a paper was in possession of the section but on account of its general character it was hardly appropriate for reading before the Association.

It was moved to adopt the resolution recommended in the report on "Dental Education," and after some amendments it was adopted, viz.:

"*Resolved*, That the interests of the profession and advanced dental education both demand that all educational institutions shall require that every student before being admitted to examination for the degree of doctor of dental surgery shall have taken two full courses of lectures."

The resolution gave rise to a free discussion in which Drs. Barrett, Stockton, Buckingham, Peirce, Markeim and Allport took part.

The resolution, as amended, was unanimously adopted.

Dr. Peirce then offered the following, which was adopted unanimously:

*Resolved*, That the American Dental Association deems it adverse to the interest of the dental profession for any State Board of Examiners to confer a title or degree of any nature."

In further discussion of the subject, Dr. Buckingham said that the Boards of Examiners should be made up outside of any college faculty; that no member of a college faculty should be a member of an examining Board.

Dr. Rehwinkel remarked that the difference between a college diploma and a certificate from an Examining Board was very great.

Dr. Peirce expressed the opinion that the time would ere long come when Examining Boards would be obsolete.

Dr. Allport thought it would be a long time before, if ever, that this would be the case.

The order of business was now suspended to hear the report of the Committee on the death of Dr. W. H. Goddard. Dr. Rehwinkel then read the report as follows:

### IN MEMORIAM.

The chair of our President is draped with the insigna of grief and mourning, indicating that its proper occupant is not with us, and that this Association has met with bereavement.

Dr. William H. Goddard, President of this Association, is no more. He died at his home in the city of Louisville on the morning of March 4, 1883, after a prolonged illness and great suffering.

Aside from the fact that it is the first officer of this Association, whose loss we mourn, the character of our friend and colleague—as a man—was such that this Association simply honors itself by giving expression to its feelings of sincere and heartfelt sorrow at his death, profound respect for and appreciation of his many noble and manly traits of character. He was the personification of honor and integrity; conscientious and exact—even to apparent sternness, in the fulfilment of duties either assigned to him or voluntarily assumed; modest and unpretending in all his stations of life, yet possessed of that manly independence of thought and opinion which enable him to become on important occasions a valuable counselor. Among his peers he was positive and strong in asserting his convictions, yet never in an arrogant or overbearing manner. In his exterior, our friend was not endowed by nature with that smooth and polished suavity of manner and address which attracts and charms at first sight, yet his excellence and strength of character soon won for him friends and honor. With all his apparent sternness of manner, he was at heart exceedingly kind and gentle. Honors came to him unsought, and whatever stations of life he occupied, or trusts administered, he was honest and faithful. His death is mourned by many who have lost in him a guardian, trustee, adviser or friend. In his family circle and among his more intimate friends, he was kind and affectionate. He was at times quite humorous, and enjoyed an innocent practical joke right well.

We all know what he was to the American Dental Association. For fourteen successive years its treasurer, until finally called forth to take the gravel and be its President, many of us have reason to remember him as an impartial but exact and unflinching officer. In his profession he strove to be abreast of the times. There was no standing still or retrograde movement with him. He was always up and doing. His career in life has been an interesting and beautiful one. It furnished material for an extended biography which has been written by able and loving friends. In token of our affectionate regard for our departed father, let a page of our records receive this our memorial, and the expression of the sincerest sorrow of this Association at his demise. Let his widow and family receive the assurance of our sympathy and condolence with them in their bereavement and distress, and our best wishes for their future welfare.

J. TAFT,
F. H. REHWINKEL,
G. W. MCELHANY,
Committee.

On motion of Dr. McKellop, of St. Louis, it was voted to inscribe the resolution, upon a memorial page in the transactions, and to send a copy to the family of the late Dr. Goddard. The vote was taken by rising.

Dr. F. M. Odell presented the following on the death of Dr. William H. Allen, of New York:

*Whereas,* We are called upon to mourn the loss of our estimable friend and former President of this Association, and co-member, it is therefore

*Resolved,* That in the demise of William H. Allen we have sustained a great loss, and that we sincerely sympathize with his family in this visitation; and as a further tribute of respect, that a page to be set aside in the records of this society as a memorial of our respect and esteem, and a copy be forwarded to the family of thc deceased.

This resolution was also passed unanimously by a rising vote.

The next business in order was the reading of the report of Section 3, "Dental Literature and Nomenclature." Dr.

J. Taft, of Cincinnati, Chairman. On motion the report was received. Dr. Atkinson then read a paper and Dr. Taft followed with a paper written by the acting president, Dr. Frederichs. Dr. Taft stated that he had mislaid a paper by Dr. Francis, but that he would forward the same for the general report. On motion it was decided to forward the paper to the publication committee for its consideration. The section was then declared open for discussion. Dr. Crouse, of Chicago, stated that there were not more than fifteen thousand practicing dentists in the United States, and he was in favor of weeding out many of the dental journals ; that one good journal containing facts and a condensed mass of literature boiled down was just what is wanted. Dr, Abbott stated that nearly all dentists were now taking dental journals, and that the desire for dental literature was on the increase, Dr, Moore, of Columbia, South Carolina, aid that he was appointed to compile a list of dentists, but it was found to be almost impossible to get a correct list, The section was then passed, and the Association adjourned until 8 o'clock P. M.

THIRD DAY—EVENING SESSION.

The Association was called together, President Frederichs in the chair.

Dr. Peirce, chairman of the committee on place of next meeting, reported Washington, St. Louis and Saratoga. The Association proceeded to select the place by ballot. On the second ballot Saratoga was chosen, receiving twenty-nine out of fifty-four votes. The following officers were then elected :

President—Dr, E. T. Darby, Philadelphia,
First Vice-President—Dr. Stockton, New Jersey.
Second Vice-President—Dr. T. F. Moore, South Carolina,
Corresponding Secretary—Dr. A. W. Harlan, Chicago.
Recording Secretary—Dr. George H. Cushing, Chicago.
Treasurer—Dr. Gorge W. Keely, Ohio.

Executive Committee—Drs. A. G. Frederichs, New Orleans ; S. G. Perry, New York ; W. N. Morrison, St. Louis.

Adjourned till 9 A. M., to-morrow.

FOURTH DAY—MORNING SESSION.

The Association was called to order at 9:30 A. M. President Frederichs in the chair.

The minutes of the last meeting were read and approved.

On motion the officers elect were installed.

Upon taking the chair, Dr. Darby addressed the Association in a very felicitous manner, extending thanks for the honor conferred and pledging his best efforts for the interest of the society, etc., etc.

Miscellaneous business now being in order, upon motion it was unanimously voted to increase the salary of the Secretary from $100 to $200.

The regular order was now resumed, and Dr. Darby, chairman of Section 4, "Operative Dentistry," read an interesting report, when the subject was discussed by Drs. Horton, Watkins, Stockton, Perry, Buckingham, and others. The use of gold in connection with baser metals was discussed by Drs. Perry, Marklain, Priest and Watkins.

The section was now passed.

On motion it was resolved that the vote passed in regard to the paper offered for the prize be reconsidered. Adopted.

The Treasurer, Dr. G. W. Keely, now presented his report which was as follows:

| | |
|---|---|
| Balance on hand August 5, 1883 | $1,446 60 |
| Received since last meeting, | 95 00 |
| Received dues this year, | 635 00 |
| Total, | $2,176 60 |
| Expenses | 528 90 |
| Balance on hand | $1,647 70 |

Drs. Rhein and Odell, of New York, and C. F. Rich, of Saratoga, were appointed as local committee.

As Committee of Publication, Drs. George Cushing, of Chicago, S. G. Perry, of New York, and A. W. Harlan, of Saratoga, were appointed by the Secretary.

A vote of thanks was extended to the retiring President, to the Secretary, and to Dr. Gates, of Niagara Falls, for assistance to the Committee on Arrangements, and also to the proprietors of hotels, to the editors and reporters of the papers who have published the proceedings of the body, and also to the railroad companies.

After the reading of the minutes of morning session, the Association adjourned to meet at Saratoga on the first Tuesday of August, 1884.—*Dental Register.*

